# Antiquorum sensing, antibiofilm formation and cytotoxicity activity of commonly used medicinal plants by inhabitants of Borabu sub-county, Nyamira County, Kenya

**DOI:** 10.1371/journal.pone.0185722

**Published:** 2017-11-01

**Authors:** Eric Omori Omwenga, Andreas Hensel, Susana Pereira, Alfred Anakalo Shitandi, Francisco M. Goycoolea

**Affiliations:** 1 Kisii University, School of Health Sciences, Kisii, Kenya; 2 University of Münster, Institute of Plant Biotechnology and Biology, Nanobiotechnology Group, Münster, Germany; 3 University of Münster, Institute of Pharmaceutical Biology and Phytochemistry, Münster, Germany; 4 Kisii University, Faculty of Applied Sciences, Kisii, Kenya; 5 School of Food Science and Nutrition. University of Leeds. Leeds, United Kingdom; Institute of medical research and medicinal plant studies, CAMEROON

## Abstract

Productions of various bacterial traits like production of virulence factors (e.g. toxins, enzymes), biofilm formation, luminescence among others, have been known to be controlled by *quorum sensing* (QS), a process that is dependent on chemical signals or autoinducers (AIs). Bacteria known to rely on such AIs are known to be virulent and tend to be resistant against various antimicrobial agents. Therefore, strategies aimed at the inhibition of QS pathways, are regarded as potential novel therapies in managing bacterial virulence hence reducing their ability to induce infections in humans. In the present study, a portfolio of 25 medicinal plant extracts (ethanol 50% v/v) used in southwestern Kenya were assayed against a transformed *E*. *coli* Top 10 reporter QS strain. This biosensor responds to the exogenous addition of 3-oxo-N-hexanoyl homoserine lactone (3OC_6_HSL) expressing green fluorescent protein (GFP). The large majority of the screened medicinal plants seemed to exhibit toxic effects and almost none of them induced antiquorum sensing (AQS) activity. This could be the consequence of the presence of mixed compounds in the extracts. *Elaeodendron buchananii* Loes and *Acacia gerrardii* Benth extracts that seemed to show AQS activity were further proved found to possess mild AQS but with defined antimicrobial activities, and no antibiofilm formation inhibition. As a control, an *E*. *coli* pBCA9145_jtk2828::sfGFP strain that produces constitutively GFP was used and confirmed that none of the two extracts quenched the fluorescence of sfGFP. Cytotoxicity assays with mammalian MDCK cells also did indicate that the selected extracts with putative AQS activity, also reduced the cell viability. Therefore, further studies will be needed to separate and re-test the individual compounds especially from the selected two promising plants.

## Introduction

It is widely agreed that quorum sensing (QS) is a mechanism of bacterial cell-cell communication that relies on the production, release, and group-wide detection of extracellular signal molecules called autoinducers (AIs). Quorum sensing (QS) enables populations of bacteria, once they grow to a critical threshold, to coordinate changes in gene expression which in most cases are geared towards biofilm formation, bioluminescence and secreting various virulence factors that determines the bacterial pathogenesis [1]. This regulatory mechanism enables bacteria to make collective decisions with respect to the expression of a specific set of genes [2]. Emerging research over the last decade or so, suggests that QS does regulate bacterial pathogenesis by controlling competence development, sporulation, antibiotic synthesis, virulence factor induction, cell differentiation, and nutrient flux along with other physiological events in pathogenic bacterial infections [3, 4, 5, 6].

QS has been found to be in some bacteria as a regulatory process to ensure the presence of sufficient cell density before a specific gene product is made. This process initiates the expression of specific phenotypes such as biofilm formation, bioluminescence, presence of flagella, synthesis of specific virulence traits like enzymes, toxins, etc. It is a process that is controlled by chemical signals called autoinducers (AI). Excess concentrations of autoinducer compounds beyond a given critical threshold within the cells are known to activate (or repress) a regulatory protein that binds to specific DNA sequence regions and activates transcription mechanism resulting in the production of such phenotypes [6, 7, 8]. The most widely studied AI compounds include fatty acid derivatives, generally N-acylhomoserine lactones (AHLs) used by Gram-negative bacteria and a furanosyl borate diester autoinducer-2 (AI-2), which is used by bacteria for interspecies communication [2]. However, other molecules such as autoinducer-3 (AI-3), which activates enterohemorrhagic *Escherichia coli* (EHEC) virulence genes, and amino acids and short peptides used by Gram-positive bacteria have also been identified as signalling molecules [2]. QS signalling molecules serve either inter- or intra-bacteria species communication but can also modulate the host immune responses.

In different bacteria species, the QS pathways are regulated by one type of AI, like AI-2 in *S*. *aureus*, while in other, more than one circuit operate, like *Escherichia coli* O157:H7 and *Salmonella typhimurium* that are known to respond to two types of auto-inducers (*e*.*g*., acylated homoserine lactones and furanosyl borate diester) [9, 10]. Marine bacterium *Vibrio harveyi* is commonly utilized as a bioluminescent reporter strain to detect the presence of autoinducer molecules at extremely low concentrations (< 1 nM) [11]. Multiple QS systems that impinge on the activity of a shared transcriptional regulator have also been reported in many bacterial species. Each of the QS systems encodes a specific receptor and AI production gene with no or limited crosstalk [12]. In several species, such as *B*. *subtilis* [13], *V*. *harveyi* [14], and its pathogenic relative, *V*. *cholerae* [15, 16], the QS pathways are arranged as in parallel, seemingly redundant, circuit architecture. That is, all the QS autoinducer receptors channel information into the same signal transduction pathway. It is so far unclear what the adaptive benefit is of harbouring multiple, rather than a single, QS autoinducer-receptor pair when the pairs function in parallel [17].

Existing studies suggest that targeting QS-regulated processes could be a new strategy for fighting bacterial infections associated to biofilm production and other virulence traits [18, 19]. Nazzaro *et al*., [19] further indicate that continued research is necessary to discover novel antimicrobial and antipathogenic agents focused on exploiting the fact that plants, surviving in an environment with a high bacterial density, may possess protective mechanisms for combating infections that could have therapeutic potential. On the other hand, it has been hypothesized that alternative strategies to the use of antibiotics, like QS-inhibition to reduce virulence and disarm the pathogens rather than killing them, could be key in eradicating the pathogens as it applies a milder evolutionary pressure that may not select for resistant strains, currently a major issue in global health challenges [1]. Motivated by these facts, on herbal and other natural products in the pursuit for new therapeutic agents has burgeoned in the recent decades.

It has been well documented that despite 10% of all terrestrial flowering plants have been used by different communities for treating diseases, only approximately 1% have gained scientific recognition and validation [19, 20]. Thus, phytochemicals may represent the richest available reservoir of novel therapeutics [21]. Although the antimicrobial activities of plant extracts are beyond doubt, in many instances, their exact mechanism of antimicrobial action is not well understood [19, 22].

This study complements our previous work focused on the ethnobotanical study of traditional medicinal plants used by local healers of the Kisii community in the Borabu sub- county in Kenya to treat gastrointestinal tract, urinary tract, skin and oral cavity [23]. Therefore in the present report, we have screened the activity of the portfolio of 25 plant extracts (ethanol 50% v/v) against an *E*. *coli* Top 10 QS reporter strain. Also, the effect of the plant extracts on the biofilm formation by the QS reporter strain and the cytotoxicity against a mammalian cell line, namely MDCK were investigated.

## Materials and methods

### Materials

Luria-Bertani bacteria culture broth, and 3-oxo-N-hexanoyl homoserine lactone (3OC_6_HSL, or generically denoted as AHL) and all other analytical grade chemicals were purchased from Sigma–Aldrich Chemie GmbH (Hamburg, Germany). M9 minimal medium was purchased from Becton, Dickinson and Company (Germany) and ampicillin (AppliChem GmbH, Germany).

### Plant materials

The plant materials were collected from Borabu sub-county (0°45′S 35°00′ E/0.750°S 35.000°E), one of the sub-counties of Nyamira County in Kenya. The portfolio of 25 plant samples (S1 Table) [23] were harvested and air dried at room temperature (25°C) under a shade. The plant roots and hard barks were chopped into small pieces prior to grounding into fine powder using a laboratory grinding mill (IKA M20-Staufen, Germany). Other dry plant parts like the leaves and some soft barks were easily ground into powder as documented (S1 Table).

### Preparation of plant extracts

Five grams of the ground powder of each plant sample was mixed with 20 mL of 50% ethanol (v/v) and was shaken for 10 min by use of a laboratory rolling mixer (IKA® Roller 6 digital) at 80 rpm. Then the mixture was placed in an Ultrasoner (Sonorex Bandelin RK-100) for 30 min as it facilitates and speeds up the extraction of organic and inorganic compounds, before it was centrifuged at 5000 rpm. The supernatant was sucked carefully by use of a micropipette and filtered unto the round bottomed volumetric flask whose weight was taken before. The same procedure was repeated on the pellet 2 times. Then the solvent in the supernatant was evaporated by use of a rotarevaporator (Büchi Rotavapor R-210) until its volume decreased drastically to ~5–10 mL. Then it was deep-frozen with liquid nitrogen and freeze-dried overnight in a Christ Alpha freeze drier unit (Model 1–4 LD plus, Martin Christ-Harz, Germany). The flask with the dried powder was weighed, removed and packed in vials until subsequent use in bioassays. The amount of extracted powder was used to calculate the yield of extraction (S1 Table). One miligram of the each extract was accurately weighed, mixed with 1 mL of 50% (v/v) ethanol and this was further diluted to come up with 0.5mg/mL. The two concentrations (1&0.5mg/mL) were used for carrying out the AQS bioassays. Those extracts with promising preliminary QS activity were further extracted using different solvents including petrol ether, dichloromethane, ethyl acetate, 99.6% ethanol, 50% (v/v) ethanol, pure methanol and cold Milli-Q water. This strategy aimed to explore thoroughly the antimicrobial and antiquorum sensing activity of a much broader range of compounds of varying solubility and polarity bound to be extracted in the different solvents.

### Biological assays

#### Bacterial strains

The bacterial strain used for all experiments was a fluorescence biosensor constructed from an *E*. *coli* Top 10 (Invitrogen, Life Technologies Co., UK), which had been transformed chemically by Celina Vila of our laboratory to contain the standard biological part BioBrick _T9002 on the plasmid BBa pSB1A3 (http://partsregistry.org/Part:BBa_T9002), kindly donated by Prof. Anderson Lab (UC Berkeley, USA). The sequence BBa T9002, comprised the *luxR* gene, coding for the transcriptional factor LuxR, under the control of the pTetR promoter, being expressed in a constitutive manner. Upon external addition of 3OC_6_HSL, the dimerization of two monomeric species of LuxR, each bound to one AHL molecule, drives to activation of *gfp* expression through binding of the LuxR-AHL dimerized complex to the lux pR promoter from *Vibrio fischeri*, and initiates the production of green fluorescent protein when 3OC_6_HSL is added [24]. This strain has been used in other QS screening studies in our laboratory [25]. The other *E*. *coli* Top10 strain was transformed with plasmid pBCA9145-jtK2828::sfGFP carrying the super folder version of the *gfp* gene kindly donated by Prof. Anderson Lab (UC Berkeley, USA) and used in control experiments. This strain expresses sfGFP constitutively from the pTetR promoter [26].

#### Antiquorum sensing assays

AHL stock solution Preparation. A stock solution 100 mM of 3OC6HSL was made by dissolving the crystalline powder in acetonitrile and placed in a vial that was tightly closed and stored at– 20°C prior to its usage. The stock solution was further diluted in sterile milliQ water to a concentration of 10 nM prior to usage in QS assays.

Bacteria suspensions preparation. A flask with 10 mL of Luria Bertani (LB) broth, supplemented with 200 μg/mL ampicillin (AppliChem GmbH, Germany), was inoculated with a single colony from a freshly streaked plate of the E. coli strain Top10 pSB1A3—BBa T9002. The test strain is resistant to ampicillin and hence the antibiotic was used to make the media to be selective to the growth of this strain. After incubation for 18 h at 37°C with vigorous shaking, 0.5 mL aliquots of the overnight culture were mixed with 0.5 mL of 30% glycerol and stored at −80◦C until further usage. Prior to each experiment, 40 μL of the bacterial glycerol stock were inoculated into 20 mL of Luria Bertani (LB) medium supplemented with 200 μg/mL ampicillin and incubated overnight at 37°C. The bacterial suspension was washed three times by centrifuging at 4000 rpm for 10 min and resuspended with Milli-Q water. Finally, the suspension was sub-diluted with sterile Milli-Q water to a final OD600 = 0.2. Prior to biosensor assays, 40 μL of the bacterial glycerol stock were inoculated into 20 mL of M9 minimal medium supplemented with 0.5% casamino acids, 1 mM thiamine hydrochloride, 0.4% glucose and ampicillin (200 μg/mL) and grown until an OD600 between 0.04 and 0.07 (∼4 h). In parallel, a vial of E. coli Top 10 biosensor or E. coli pBCA9145_jtk2828::sfGFP bacteria (stored at -82°C) was taken and sub-cultured unto LB agar medium overnight at right conditions. Then one small round white colony is identified, picked aseptically and inoculated unto the 10 mL of LB broth with 10μL of ampicillin (200 mg/ml) aseptically. Overnight incubation at 37°C, 100 rpm was done. Stock vials of this test strain were then made by mixing 500 μL of the overnight cultured bacteria with 500 μL of 30% glycerol in vials and they were kept at -82°C for use in experiments.

Quorum sensing and control assays. The various concentrations of the plant extract stocks (0.5 and 1mg/mL) were made by dissolving them in 1 mL of 50% (v/v) ethanol. To each well of the flat-bottomed 96-well plate (Greiner Bio-One, cat. # M3061) were transferred 10 μL of AHL (10 nM), 10 μL of the dilute plant extract sample and 180 μL of the bacterial suspension (OD600 between ~ 0.04 and ~ 0.07). Three blank wells with 10 μL of AHL (10 nM), 10 μL of the dilute plant extract sample and 180 μL of medium were used to measure the absorbance background. M9 broth and 50% (v/v) ethanol were also used as controls of background absorbance. Three additional blanks were also included as controls of background fluorescence, namely: Blank No. 1. - 180 μL of the M9 broth and 20 μL of water; Blank No. 2–20 μL of sterile Milli-Q water and 180 μL of bacterial suspension; Blank No. 3.- 10 μL of AHL, 10 μL of sterile Milli-Q water and 180 μL of bacterial suspension. For 50% (v/v) ethanol wells comprised of 10 μL of 50% (v/v) ethanol, 10 μL of AHL and 180 μL of bacteria.

The plate was incubated in a Safire Tecan-F129013 Microplate Reader (Tecan, Crailsheim, Germany) at 37 ºC and with vigorous orbital shaking during the five seconds before each measurement. Absorbance and fluorescence were measured every six minutes with the following parameters: (fluorescence λ_ex_ = 485 nm, fluorescence λ_em_ = 520 nm, integration time = 40 μs, number of flashes = 10, gain = 100, measurement mode = top). To avoid complications due to excessive evaporation during the absorbance and fluorescence determinations, only 294 min of growth were recorded. For each experiment, the fluorescence intensity (FI) and OD_600_ data were corrected by subtracting the values of absorbance and fluorescence backgrounds and expressed as the average for each treatment.

Control assays with the *E*. *coli* pBCA9145_jtk2828::sfGFP strain expressing GFP in the absence of AHL sensing were conducted in identical conditions as explained above, but without the addition of AHL.

#### Antibiofilm formation activity

A microtiter plate assay, as reported elsewhere [27], with some modifications, was performed to quantify the effect of plant extracts in on the biofilm formation of E. coli Top 10 pSB1A3—BBa T9002 strain. The test bacteria was first of all inoculated on LB agar and incubated at right conditions at 37°C overnight. Then a colony was identified, picked and inoculated in 10 mL of LB broth and incubated at 37°C overnight at 100 rpm. An aliquot of 190 μL of M9 broth with and without the 50% (v/v) ethanol plant extracts (1 and 0.5 mg/mL) were then inoculated with 10 μL of bacterial suspension per well and incubated at 37°C for 48 h without shaking. Kanamycin (1mM) was used as a positive control and M9 broth as negative control. The flat-bottomed polystyrene tissue culture microplate was sealed with a parafilm to prevent medium evaporation. After incubation, the wells were carefully rinsed with double-distilled water to remove loosely attached cells. The microplate was air-dried for 1 h before adding 200 μL per well of 0.4% crystal violet solution to the adhered cells in the wells and it was left at room temperature for 15 min. Excess stain was then removed by rinsing the wells with 200 μL per well of distilled water three times. The microtiter plate was then air-dried again for 1 h after which 200 μl per well of absolute ethanol was added to solubilize the dye. Intensity was measured at OD590nm by using a Safire Tecan-F129013 Microplate Reader (Tecan, Crailsheim, Germany). For each experiment, background staining was corrected by subtracting the crystal violet bound to un-treated controls (Blank) from those of the tested sample. The experiments were done in triplicate and average OD590nm values were calculated. To estimate the antibiofilm activity (Abf A) of a given extract the following equation was used

$$Abf\;A\;(\%)=[1-\left({OD}_{Test\;sample}-{OD}_{Blank}\right)/\left({OD}_{Untreated\;sample}-{OD}_{Blank}\right)]\times 100$$

#### Colony forming units (cfu) assay

A culture was prepared with 20 mL LB broth, 20 μL ampicillin (1000x, 200μg/mL, AppliChem, Darmstadt) and 40 μL *E*. *coli* Top 10 pSB1A3—BBa T9002 glycerol stock. It was incubated overnight at 37°C with shaking at 100 rpm. The OD_600_ was adjusted to 0.1 and a microtiter plate with 180 μL of the diluted *E*. *coli* Top 10 pSB1A3—BBa T9002 culture and 20 μl of both 1 mg/mL and 0.5 mg/mL of the methanol plant extract was incubated at 37°C and 100 rpm for 1 h. One millimolar (mM) of kanamycin antibiotic was used as a positive control, 50% ethanol and LB broth as untreated controls. Afterwards, a serial dilution was performed in a 96-well-plate in 7 steps with 180 μl M9 medium each until a dilution factor of 10^−7^ was reached. Then, all the dilutions were dropped onto LB-ampicillin-agar-plates in triplicate in drops of 10 μl. The plates were incubated overnight at 37°C and the colonies were counted the next day. A weighed average was calculated from the dilutions.

#### MTT cell viability assay on MDCK-C7 mammalian cells

Cell culture. Mandin Darby Canine Kidney (MDCK) cells clone 7 (C7) were kindly provided by Prof. Dr. W. Schneider of clinic of dermatology Mannheim University (Germany). The MDCK-C7 were cultured in 75 cm^2^ flasks using MEM supplemented with 10% fetal bovine serum, 1% L-glutamine (200 mM) and 1% penicillin-streptomycin (10000 units penicillin, 10000 units streptomycin in 0.9% NaCl) [28]. The cell cultures were maintained in a humid atmosphere at 37°C with 5% CO2 (Sanyo MCO-19AIC, Panasonic Biomedical Sales Europe BV, AZ Etten Leur, Netherlands). After reaching microscopic confluence, the cells were washed with 10 ml phosphate buffered saline (PBS) and trypsinized with 10 ml 0.05% trypsin in EDTA (1x) buffer. After detachment, 10 ml of MEM was added to the trypsin buffer. The cell suspension was centrifuged at 1000 r.p.m for 5 min (Rotina 420 R, Hettich GmbH, and Tuttlingen, Germany). The excess of medium was removed by sucking and the cell pellet was resuspended in 1 ml MEM. A 10-μl aliquot of the cell suspension was diluted with 90 μl trypan blue and the number of cells was counted with an improved Neubauer chamber before seeding. The cells were subcultured by splitting at a ratio of 1:10 [28].

The influence of the studied plant extracts on the cell viability was studied using a model mammalian cell line, namely the Madin-Darby canine kidney (MDCK-C7) using the well-known MTT assay [29, 30]. This test is indicative for mitochondrial NADH-dependent dehydrogenase activity, which is directly proportional to metabolic capability and it is used as an indicator of cellular proliferation. Aliquots of 100 μL of a cell suspension were seeded in a 96-well polystyrene tissue culture plates at a density of 5 x 10^⁵^ cells mL^-1^ using minimum essential medium eagle (MEM) containing 1% penicillin/streptomycin, supplemented with 10% fetal bovine serum (FBS). The plates were cultured in a humidified atmosphere of 5% CO_2_ at 37°C. After 24 h, the wells were washed twice with 100 μL/well of MEM without supplements to get rid of the unattached cells. Then a 100 μL/well aliquot of the plant extract dissolved in nonsupplemented MEM was added to the well at the varying concentrations (0.01, 0.025, 0.05, 0.1 and 0.25 mg/mL). As a positive control, it was used 4% Triton X-100. The cells were then further incubated for additional 24 h at 37°C in a humidified atmosphere of 5% CO_2_. After incubation, the plant extracts were sucked off the wells and washed again twice with 100 μL/well of nonsupplemented MEM. Then 100 μL/well of MEM without supplements and 25 μL/well of MTT (5 mg/mL of thiazolyl blue tetrazolium bromide) were added and incubated for 4 h at 37°C. This was followed by removal of non-supplemented MEM and the MTT mixture and 100 μL aliquots of dimethyl sulfoxide (DMSO) were added to each well to dissolve the formazan crystals, followed by incubation for 10 min to dissolve air bubbles while shaking at 100 rpm. The culture plate absorbance was then measured at λ = 590 nm on the Safire—Tecan F129013 micro-plate reader instrument (Tecan, Crailsheim, Germany) used for the previous biological assays. The amount of colour produced is directly proportional to the number of viable cells. Cell viability (%) was calculated using equation [31]:

$$Cell\;viability\;(\%)=A_{test\;wells}/A_{control\;wells}\times 100$$

### Data analysis

All biological experiments were carried out in triplicates to validate reproducibility and data were presented as mean ± S.E of the mean. The graphs were constructed using Graph Pad Prism software (version 6.01; Graph Pad Software, Inc., La Jolla, CA, USA). One-way analysis of variance was performed to compare differences between groups. P-values less than 0.05 were considered significant.

## Results

### Screening of antiquorum sensing activity of plant extracts against an *E*. *coli* Top 10 biosensor

**Fig 1:**
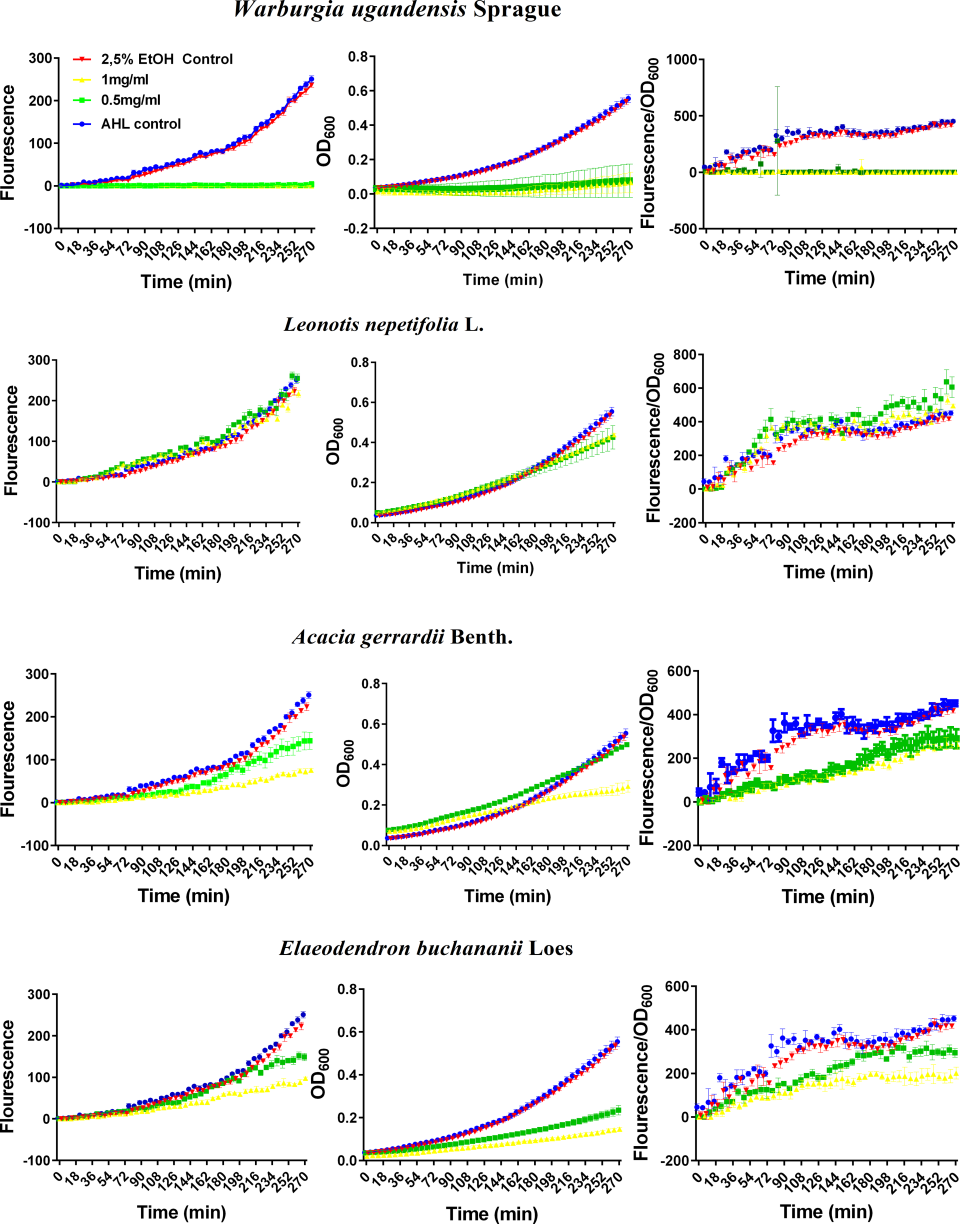
Representative plots of the evolution of the fluorescence (left panels), growth–as OD_600_- (center panels) and fluorescence intensity corrected for growth (fluorescence/OD_600_) (right panels) of *E*. *coli* strain Top 10 transformed with plasmid pSB1A3—BBa_T9002 after treatment with ethanol 50% (v/v) extracts of *Warburgia ugandensis* Sprague (Code P20), *Leonotis nepetifolia* L. (Code P9), *Acacia gerrardii* Benth. (Code P22) and *Elaeodendron buchananii* Loes (Code P21) applied at 0.5 and 1.0 mg/mL (as per legend in the top-left panel). The *E*. *coli* quorum sensing response was induced by the addition of (3OC_6_HSL) to a final concentration of 0.5 nM. OD_600_ and fluorescence intensity were measured every six minutes with the appropriate parameters (fluorescence λ_ex_ = 485 nm, fluorescence λ_em_ = 520 nm, integration time = 40 μs, number of flashes = 10, gain = 100, measurement mode = top). Data are mean values ± SD (*n* = 3).

**Fig 2:**
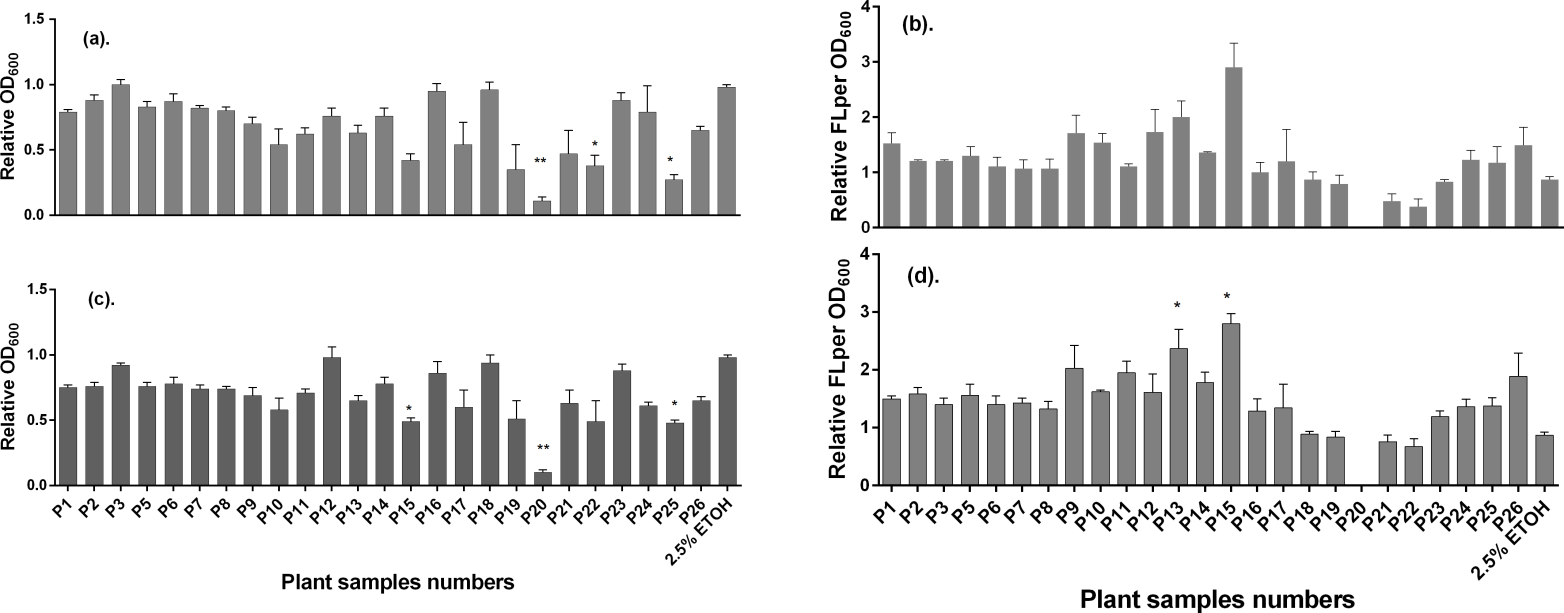
**Summarized bioactivity of the 25 screened plant extracts about growth (OD**_**600**_**) and quorum sensing (FL/OD**_**600**_**) response of the *E*. *coli* strain Top 10 transformed with plasmid pSB1A3—BBa_T9002 after treatment with ethanol 50% (v/v) extracts at concentrations of: 1.0 mg/mL (a) and (b), and 0.5 mg/mL (c) and (d) relative to the untreated control, as recorded during the last 294 min of incubation at 37°C.** Key: Code P1 (*Oxalis corniculata* L.), Code P2 (*Urtica dioica* L.), Code P3 (*Erlangea marginata* S. Moore), Code P5 (*Rubia cordifolia* L.), Code P6 (*Spilanthes mauritiana* DC.), Code P7 (*Orthosiphon hildebrandtii* Vatke), Code P8 (*Dichrocephala integrifolia* Kuntze), Code P9 (*Leonotis nepetifolia* (L) R. Br.), Code P10 (*Rhoicissus tridentata* (L.f) Wild & Drumm), Code P11 (*Toddalia asiatica* (L.) Lam), Code P12 (*Asparagus africanus* Lam.), Code P13 (*Balanites orbicularis* Sprague), Code P14 (*Clerodendrum myricoides* (Hochst) R.Br. & Vatke), Code P15 (*Caesalpinia decapetala* (Roth) Alston), Code P16 (*Solanum renschii* Vatke), Code P17 (*Croton macrostachyus* Hochst. ex Delile), Code P18 (*Erythrina abyssinica* Lam.), Code P19 (*Rhus natalensis* Bernh. Ex Krauss), Code P20 (*Warburgia ugandensis* Sprague), Code P21 (*Elaeodendron buchananii* Loes), Code P22 (*Acacia gerrardii* Benth.), Code P23 (*Carissa edulis* Vahl), Code P24 (*Cassia didymobotrya* Fresen, Code P25 (*Acacia nilotica* (L.) Delile.) and Code P26 (*Bischofia javanica* Blume). Data are mean values ± SD. Statistical test: Kruskal-Wallis test (*n* = 3, *p<0.01, **p<0.05, ***p<0.001).

The results of the biological assays as examined with the *E*. *coli* Top 10 QS reporter strain indicated that most of the plant extracts (see S1 Table) from the portfolio exhibited antimicrobial activities. This was evidenced from the growth curves during incubation at 37°C. Based on the magnitude of this antibacterial activity, therefore, the plant extracts were classified into three main categories, namely: i) Plant extracts with full antimicrobial activity (*i*.*e*., those that maintained the OD_600_ at minimum values due to total inhibition of the growth or eradication of the bacteria). In this category, we found the extract of *Warburgia ugandensis* Sprague, Code P20 (Fig 1); ii) Plant extracts with negligible effect neither on growth (*i*.*e*., OD_600_) nor on QS inhibition (*i*.*e*., fluorescence intensity/OD_600_). These include extracts of plants such as *Leonotis nepetifolia* (L) R. Br. (Code P9, Fig 1), *Cassia didymobotrya* Fresen Code P24, *Rhoicissus tridentata* (L.f) Wild & Drumm Code P10 amongst others. iii) Plant extracts with moderate effect on growth inhibition (*i*.*e*., OD_600_) but clear effect on QS inhibition (*i*.*e*., fluorescence intensity/OD_600_). To this end the extracts of *Acacia gerrardii* Benth (Code P22, Fig 1) and *Elaeodendron buchananii* Loes (Code P21) were found to belong to this category. Even when it showed antimicrobial activity at the tested doses (≥0.5 mg/mL), the fluorescence corrected by OD_600_ shows a similar response to that of *Acacia gerrardii* Benth as illustrated in Fig 1. In turn, Fig 2 summarizes the activity of all the tested plant extracts expressed as relative growth (OD_600_), and relative QS (fluorescence intensity/OD_600_). To determine these two parameters, the data of the last 294 min of the OD_600_ and of fluorescence/OD_600_ curves where averaged and divided by those of the untreated control. Notice in Fig 2B and 2D that the relative QS (fluorescence intensity/OD_600_) of most of plant extracts led to values greater than 1.0 at the two tested doses (0.5 and 1.0 mg/mL), namely P1 to P17 and P24 to P26, and P23 only at the lower dose.

Only extracts of *Rhus natalensis* Bernh. Ex Krauss (Code P19), *Warburgia ugandensis* Sprague (Code P20), *Elaeodendron buchananii* Loes (Code P21) and *Acacia gerrardii* Benth. (Code P22) showed a significant decrease in the relative QS response corrected by growth. We selected the extracts of *Acacia gerrardii* Benth (Code P22) and *Elaeodendron buchananii* Loes (Code P21), that led to much decrease in QS and a moderate reduction in growth for subsequent studies, as they did not inhibit bacterial growth (OD reduction) as much as *Rhus natalensis* Bernh. Ex Krauss (Code P19) and *Warburgia ugandensis* Sprague (Code P20) did.

### Antiquorum sensing activity of selected plant extracts against *E*. *coli* Top 10 and *E*. *coli pBCA9145_jtk2828*::*sfGFP*

**Fig 3:**
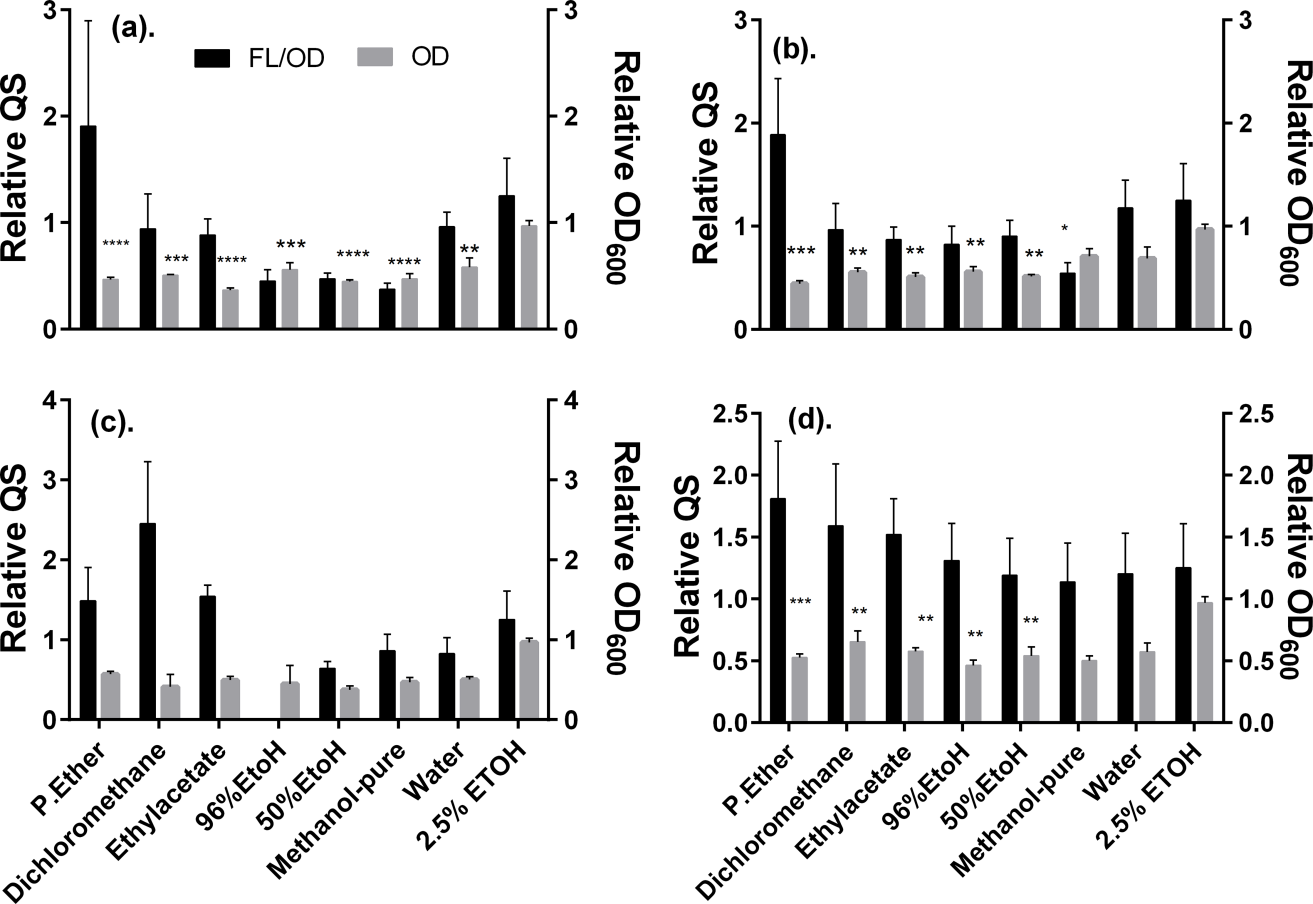
Summarized bioactivity of the *Elaeodendron buchananii* (a) and (b*)* and *Acacia gerrardii* (c) and (d) extracts about growth (OD_600_) and quorum sensing (FL/OD_600_) response of the *E*. *coli* strain Top 10 transformed with plasmid pSB1A3—BBa_T9002 after treatment with various solvents extracts at concentrations of: 1 mg/mL (a) and (c), and 0.5 mg/mL (b) and (d) relative to the untreated control, as recorded during the last 294 min of incubation at 37°C. Data are mean values ± SD. Statistical test: One way ANOVA test (n = 3, *p<0.01, **p<0.05, ***p<0.001 & ****p<0.0001).

The relative antimicrobial and antiquorum sensing activity of the different solvent extracts of the two promising AQS plants are summarized in Fig 3. What draws attention most after a careful inspection of the plots is that the extracts obtained using different solvents led to somewhat similar reduction of the bacterial growth (OD_600_) with respect to the untreated control, and in both cases, a slight dose-dependent response was noticed (*cf*. data of 0.5 vs 1.0 mg/mL). Also, it seems clear that the extracts of *Acacia gerrardii* (Code P22), in comparison to those of *Elaeodendron buchananii* Loes (Code P21), inhibited slightly more strongly the bacterial growth. These results are consistent with the possible occurrence of different type of compounds with antibacterial activity that vary in polarity and solubility and hence occur throughout the different extracts.

**Fig 4:**
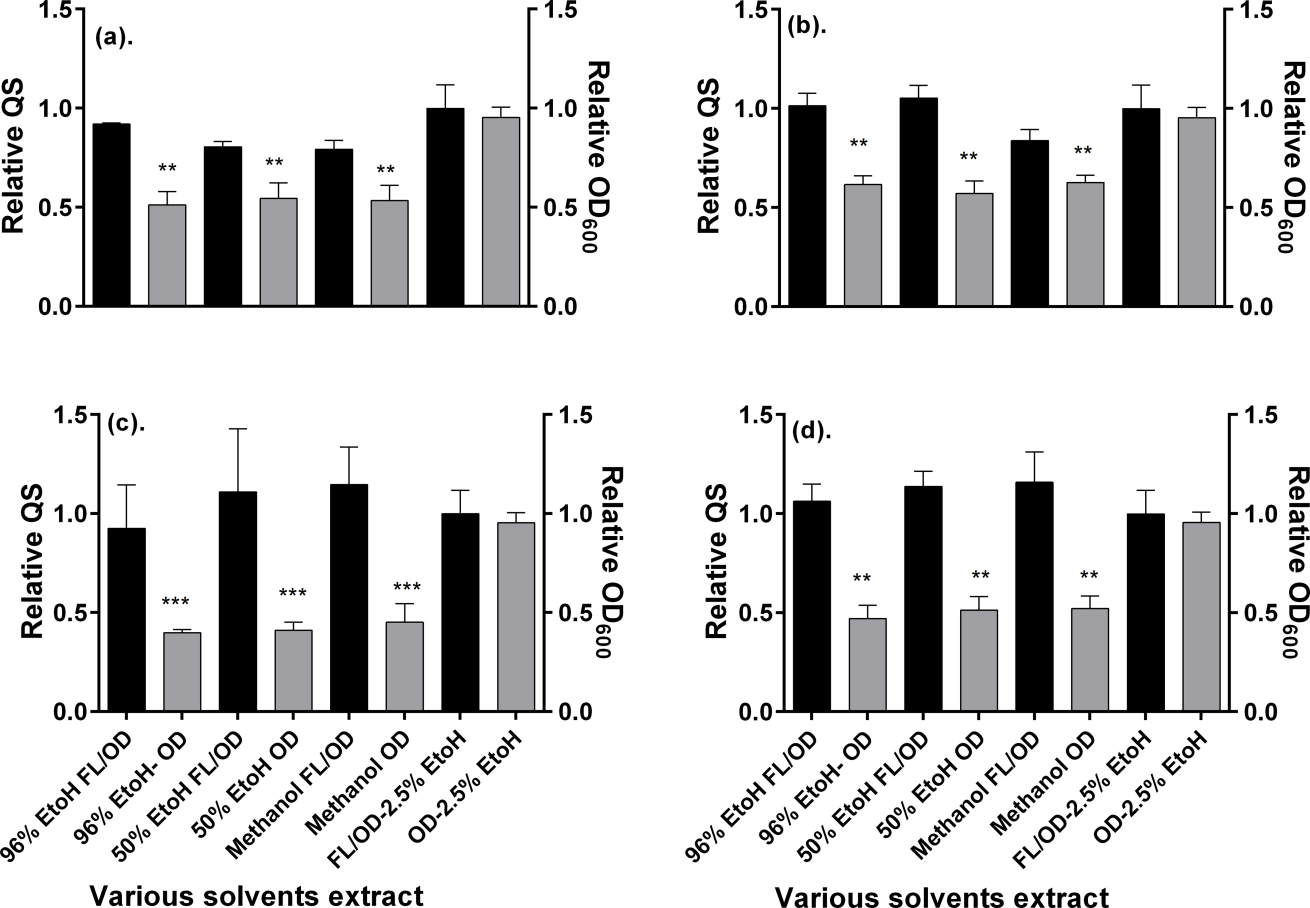
Summarized bioactivity of the *Elaeodendron buchananii* (a) and (b*)* and *Acacia gerrardii* (c) and (d) extracts about growth (OD_600_) and quorum sensing (FL/OD_600_) response of the *E*. *coli* pBCA9145_jtk2828::sfGFP after treatment with various solvents extracts at concentrations of: 1 mg/mL (a) and (c), and 0.5 mg/mL (b) and (d) relative to the untreated control, as recorded during the last 294 min of incubation at 37°C. Data are mean values ± SD. Statistical test: One way ANOVA test (n = 3, *p<0.01, **p<0.05, & ***p<0.001).

Notice that only the 99.6% ethanol, 50% ethanol, and pure methanol extracts reduced significantly the relative quorum sensing (fluorescence/OD_600_). Therefore, to examine the possible interference of the compounds present in the extracts of both plants on the quenching of the fluorescence of GFP, we tested the influence of these extracts on the *E*. *coli* pBCA9145_jtk2828::sfGFP strain. In contrast with the *E*. *coli* Top 10 QS biosensor strain, *E*. *coli pBCA9145_jtk2828*::*sfGFP* is a strain that has the ability to produce its own GFP naturally without the need of exogenous AHL. This strain serves as a control to assess any potential interference of the tested substance with the fluorescence of GFP due to quenching. Hence, it enables to distinguish true QS inhibition (mediated by AHL) from direct fluorescence quenching of the reporter GFP. The results of these assays are summarized in Fig 4 below.

It should be noticed that the results of these experiments were in agreement with those obtained with the *E*. *coli* Top 10 QS biosensor strain. It was possible to confirm that the three extracts lead to a reduction of the bacterial growth, and that relative fluorescence/OD_600_ signal does not vary too much from the expected value of 1.0. These experiments allowed ruling out any possible interference of the present compounds with the fluorescence of GFP.

### Influence of *Elaeodendron buchananii* Loes extracts on the relative growth and relative antiquorum sensing activity

**Fig 5:**
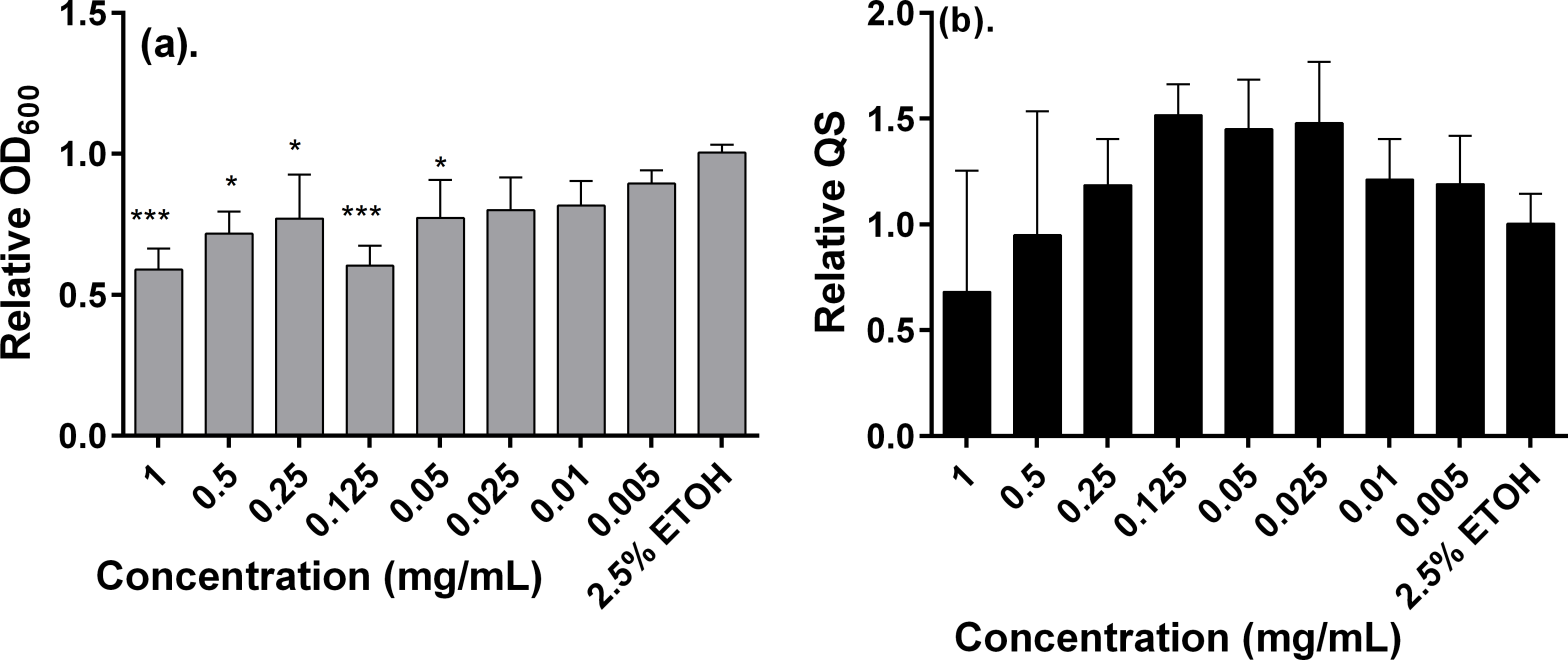
**Summarized bioactivity of various concentrations of methanol extracts of *E*. *buchananii* about growth (OD**_**600**_**) (a) and quorum sensing (fluorescence/OD**_**600**_**) (b) response of the *E*. *coli* Top10.** Data are mean values ± SD. Statistical test: One way ANOVA test (*n* = 3, *p<0.01, **p<0.05, and ***p<0.001).

Since the extracts of *Elaeodendron buchananii* Loes exhibited overall lower toxicity than did the rest of the plant extracts in addition to moderate antiquorum sensing activity, we examined in further detail the dose-dependent response of the methanol extracts on these responses. To this end, varying concentrations of the methanolic extract were assessed for the evaluation of their effect on relative cell growth (OD_600_) and relative quorum sensing activity (fluorescence/OD_600_). Fig 5 illustrates the outcome of these experiments. Notice in Fig 5A that there is a clear dose-dependent increasing linear response about the effect of decreasing concentrations of the extract ranging from 1 to 0.005 mg/mL on the relative OD_600_. A closer inspection of the plot in Fig 5B also reveals that the relative QS response decreased in a dose-dependent manner as the concentration varied from 0.05 to 1.00 mg/mL, with not much subsequent variation at lower concentrations.

With the aim to examine in further detail the effect of the methanol extracts of *Eleaodendron buchananii* Loes on the cell viability of the *E*. *coli* Top 10 QS biosensor strain, we performed an agar plate CFU determinations. The results of these assays (see S1 Fig) confirmed that there is a dose-dependent response on the antibacterial activity when compared the two assayed concentrations of the extract (namely 0.5 and 1.0 mg/mL). However, the determined numbers of CFUs were within the same order of magnitude as the untreated control, thus reflecting the low toxicity of the extracts.

### Influence of extracts of selected plant extracts on the biofilm formation inhibition of the *E*. *coli* top 10 biosensor

**Fig 6:**
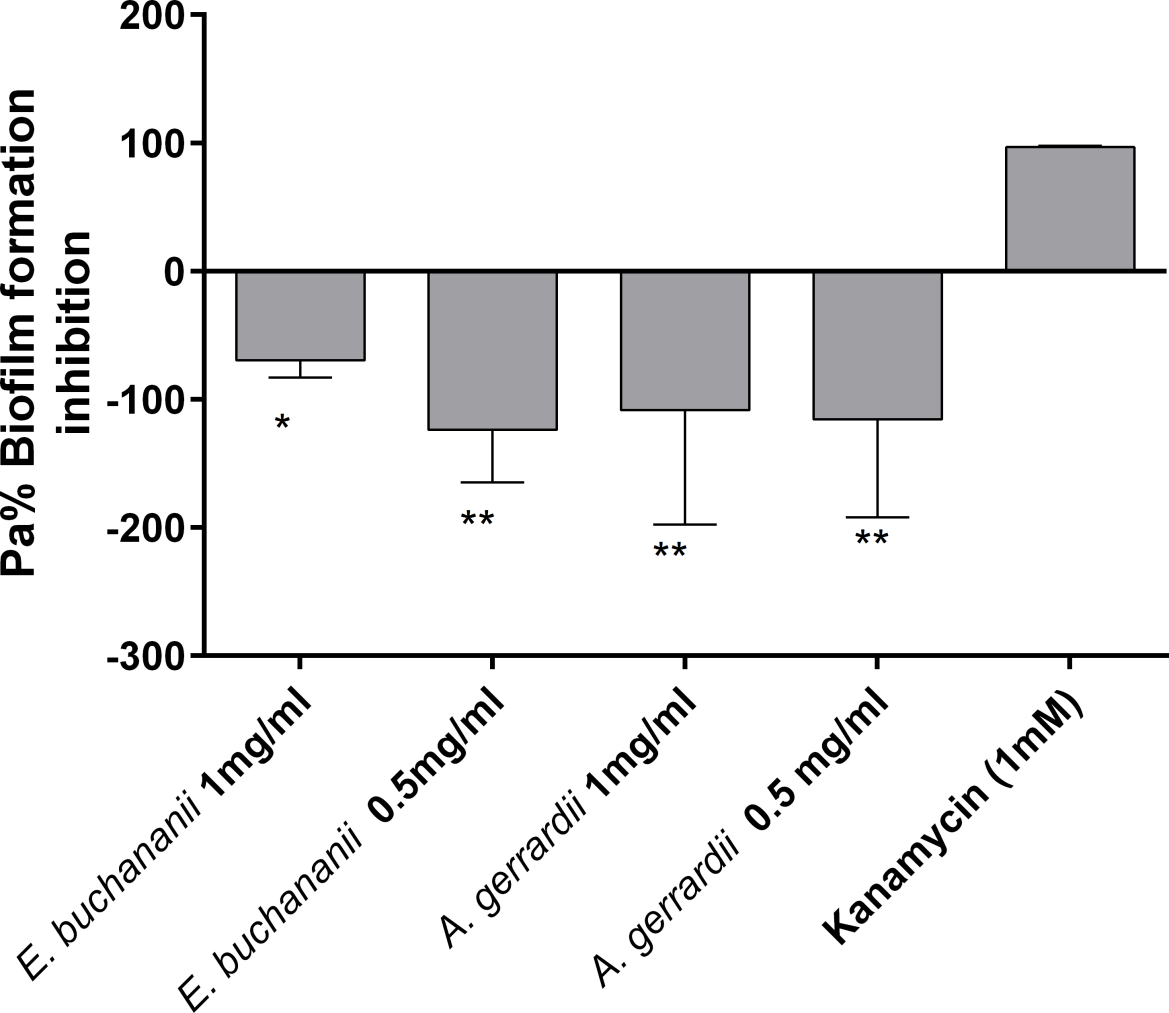
Antibiofilm activity of methanol extracts of *E*. *buchananii* and *A*. *gerrardii* against *E*.*coli* Top 10. One way ANOVA test (n = 3, *p<0.01, and **p<0.05).

An antibiofilm assay was also carried out on the pure methanol extracts of *Elaeodendron buchananii* and 50% ethanol (v/v) *Acacia gerrardii*. Neither of the two extracts showed any ability to inhibit biofilm formation as presented in Fig 6 below.

### Influence of selected plant extracts on the metabolic competence of MDCK cells

**Fig 7:**
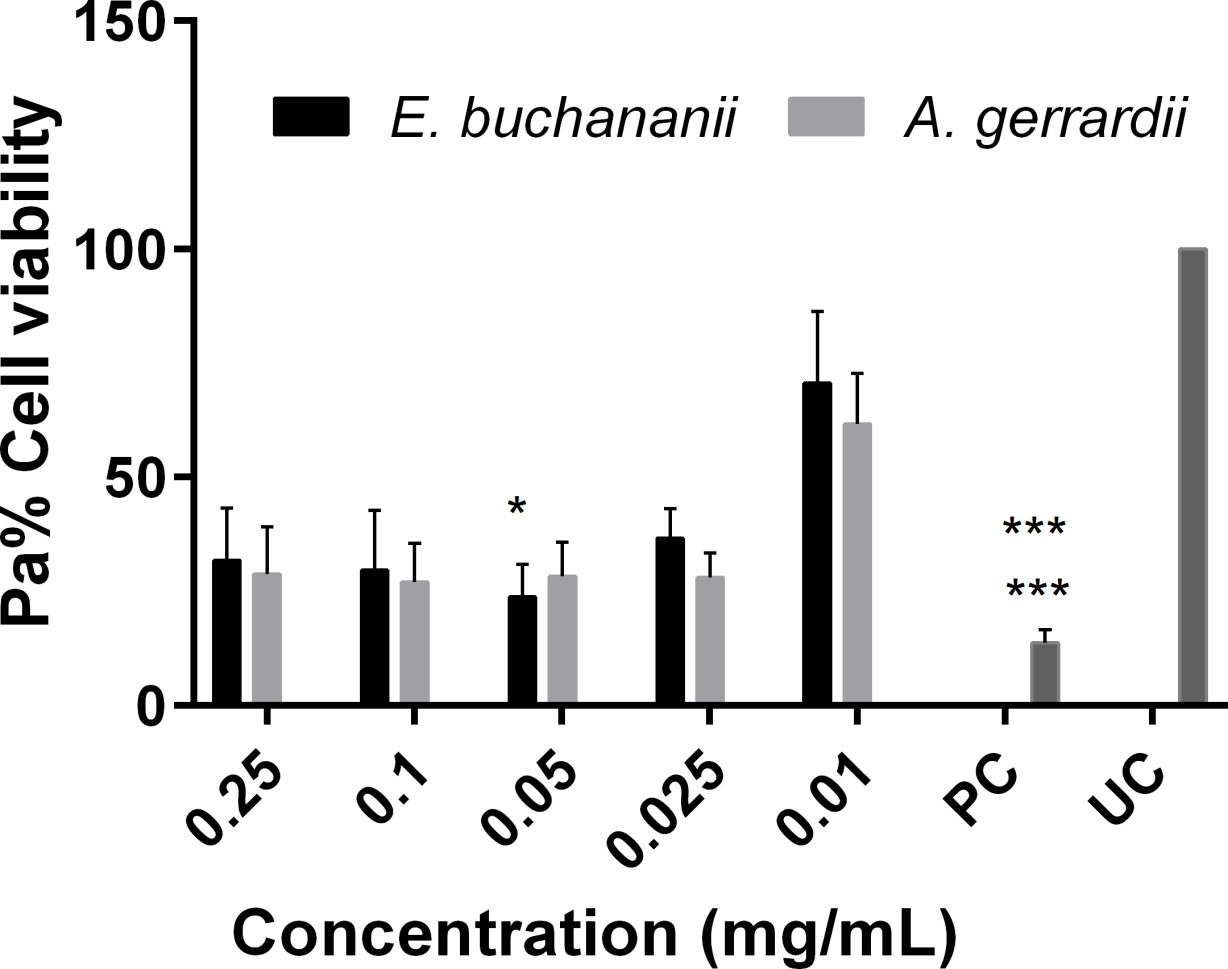
Cytotoxicity of 50% ethanol extracts of *E*. *buchananii* and *A*. *gerrardii* against MDCK-C7 cells (*n* = 3, * p < 0.05, *** p < 0.001; Friedman test and *** p < 0.001).

The cytotoxicity of the 50% (v/v) ethanol extracts of *Elaeodendron buchananii* and *Acacia gerrardii* was also determined by use of the well-known MTT assay. Both extracts showed to decrease the cell viability of the MDCK mammalian cells to < 50% when applied at concentrations ≥ 0.025 (Fig 7). It became apparent that at higher concentrations (0.25 mg/mL), there was a slight increase in cell viability. However, this may be the consequence of an artefact associated to the possibility of both extracts seem to be oxidising the MTT reagent (see S2 Fig).

## Discussion

Medicinal plants have been used for ages despite the availability of modern medicines. Many communities (either by choice or for lack of economic resources) in rural areas of Kenya still use traditional medicine as a first-line treatment and have a vast arsenal of indigenous medicinal plants which are typically prescribed by local healers [23, 32]. Medicinal plants are enormously rich in phytochemicals which can target various sites or pathways of the pathogens hence killing or inhibiting growth of the pathogen [19]. Several active components from plants offer a repertoire of antimicrobial agents and have attracted considerable scientific interest. Even though the antimicrobial nature of these compounds has been proved, neither the underlying mechanisms of their mode of action are yet fully clearly understood nor the specific individual components are known [33]. Even though reports on the antimicrobial properties of these medicinal plants phytochemicals are available, systematic studies on their QS inhibitory and antibiofilm activities are scarce. The current study, therefore, first on its kind, aimed at expanding the scientifically-based knowledge on the medicinal plants used by the Kisii community in Borabu sub county in Nyamira County–Kenya.

Nearly since the discovery of bacterial QS, targeting it has been regarded as a potential novel strategy to manage pathogenic bacteria by interfering with their virulent traits like biofilm formation, enzymes production, swarming activity, etc. Potential approaches in this regard include discovering compounds which degrade the autoinducers, interfere with the signal receptor, inhibit the synthesis of the autoinducers or sequester the ones that have been produced [34, 35]. Therefore, for a given phytochemical or compound to qualify as effective QS inhibitor, it must have no effect on the growth of the bacteria but should specifically inhibit the QS-related pathways (e.g. synthesis of autoinducers, target specific proteins, etc.) that mediate the virulent response to the host. In principle, this strategy should lead to the dismantling the pathogenic/virulent capacity of bacteria, thus rendering them susceptible for subsequent elimination by the host immune system. This approach is bound to reduce the selective pressure on bacteria and thus may reduce the levels of resistances to such compound hence its long term usage. Whether antibiotic resistance is governed by QS is yet to be known. However, since enzyme production (e.g. β-lactamases, elastases etc.) often is under QS regulation, we can anticipate that combined therapies using antibiotics and QS inhibitors may contribute to overcome resistance of certain pathogenic species. This hypothesis is yet to be validated.

Our present data on the portfolio of medicinal plants from southwest Kenya indicates that most of the medicinal plants extracts screened did have antimicrobial activities against the two *E*. *coli* strains (*E*.*coli* Top 10 QS biosensor strain and *E*. *coli* pBCA9145_jtk2828::sfGFP strain). Indeed, *Warburgia ugandensis* Sprague (Code 20) almost killed completely the bacteria at concentrations of 1 and 0.5 mg/mL. *W*. *ugandensis* has already been reported to exhibit antimicrobial properties in concurrence with our findings [36]. It is therefore clear that this extract could be having strong antimicrobial activities and future studies should focus on identifying the individual phytochemicals responsible for the strong antimicrobial potency.

Other screened extracts from *Leonotis nepetifolia* (L) R. Br. (Code P9), *Cassia didymobotrya* Fresen (Code P24), *Rhoicissus tridentata* Wild & Drumm (Code P10), amongst others, also reduced the OD_600_ of the bacteria by a quite a smaller margin and yet this did not have any inhibit GFP production by our test strain. This is a clear indication that for bacteria to express GFP, a given critical threshold of the numbers of bacteria and dimerized LuxR receptors is needed, hence quorum sensing effect [24]. Another conclusion that can be drawn from the results of this study is that none of the plant extracts seemed to display the specific inhibition of the AHL-mediated QS activity in the used *E*. *coli* Top 10 biosensor used. It has also been reported that *Cassia didymobotrya* (Code P24) shows antimicrobial activity against some Gram-positive and Gram-negative bacteria, as well as some fungal isolates in a dose dependent manner [37], in concurrence with our own results. Whether sub-lethal doses (at much lower dose than 0.5 mg/mL) of this extract could have antiquorum sensing activity is yet to be investigated. We have explored this scenario for extracts of *Elaeodendron buchananii* Loes (Code P21) at varying doses though.

In turn, extracts of *Oxalis corniculata* L. (Code P1), *Urtica dioica* L. (Code P2), *Erlangea marginata* S. Moore (Code P3), *Rubia cordifolia* L. (Code P5), *Clerodendrum myricoides* (Hochst) R.Br. & Vatke (Code P14), *Erythrina abyssinica* Lam. (Code P18), amongst others, seemed to have only a moderate antibacterial effect against the *E*. *coli* Top 10 QS biosensor, and none on the reduction of the expression of GFP, hence, no capacity of QS inhibition. Previous reports on *Clerodendrum myricoides* have, however, indicated that this medicinal plant does exhibit antimicrobial activity, [38, 39] a fact that concurs with our findings.

It is highly likely that some of the medicinal plant extracts exhibited a mixture of compounds with distinctive activities (*i*.*e*., those that have antimicrobial properties and those that inhibit QS via AHL-LuxR receptors transcriptional activation). It has been reported that some medicinal plants phytochemicals do mimic the autoinducers of quorum sensing. For instance, rosmaric acid has been proved to have AHL properties with *P*. *aeruginosa* PA01. This compound increased RhIR mediated transcription *in vitro* more than C_4_-HSL [40]. Also, the algal compound lumichrome, a riboflavin derivative, has also been reported to stimulate the activity of a QS regulator of *Pseudomonas aeruginosa* [41]. Therefore, in the extracts of plants such as *Leonotis nepetifolia* (Code P9), *Rhoicissus tridentata* (Code P10), and *Caesalpinia decapetala* (Code P15), compounds that possess both antimicrobial properties and those that mimic AHL autoinducer may co-exist.

Ethanol 50% (v/v) extracts of *Elaeodendron buchananii* Loes and *Acacia gerrardii* Benth seemed to show some promising antiquorum sensing activity, as they had reduced both the relative OD_600_ and also the relative fluorescence/OD_600_. In light of these promising results, further studies focused on these two plants were conducted, namely by exploring the bioactivity of other extracts obtained with different solvents, and by conducting control experiments with the *E*. *coli pBCA9145_jtk2828*::*sfGFP* that expresses constitutively GFP in the absence of added AHL. These later assays allowed confirming that the phytochemicals present in the different extracts of both plants did not quench themselves the fluorescence of GFP. Hence, this evidence is consistent with the suggestion that the reduction on relative FL/OD_600_ may be the consequence of the specific inhibition of QS pathways mediated by AHL and LuxR. However, the inhibition of cell growth that accompanies the putative antiquorum sensing activity does not provide compelling evidence that will allow us to draw conclusions in this regard.

In the case of both plants, it was also quite clear that the different extracts from less polar solvents like petrol ether and dichlomethane reduced the relative OD_600_ but FL/OD_600_ was elevated steadily. This is a clear indication that both *A*. *gerrardii* and *E*. *buchananii* extracts do possess both polar and less polar compounds and some of which may be having strong antimicrobial properties while others may be able to interfere with the AHL-governed QS processes. Further studies are needed focused on both extracts to deduce if these groups of compounds occur on them.

In general, the extracts of *A*. *gerrardii* (Code P22), seemed to be more toxic as compared to those of *E*. *buchananii* (Code P21). Therefore, lower concentrations of the methanol extracts of *E*. *buchananii* (Code P21) were evaluated (Fig 5) but no inhibitory effects on the GFP production. At higher doses it did indicate toxicity to the test strain and reduction in GFP production. This could be related to the fact that toxicity reduced the concentration of test strain as they must be of a given threshold for them to induce QS activity. Yet a further assay was conducted aiming to determine the influence of the methanol extracts of *E*. *buchananii* (Code P21) on the number of CFUs. A dose-dependent reduction on the value of CFU was found for the two assayed concentrations of the extracts (see S1 Fig). However, these were within the same order of magnitude as the untreated control and the corresponding 50% ethanol control. Thus, these results offer diagnostic evidence that the antibacterial activity of extracts of *E*. *buchananii* (Code P21), if the doses are correctly managed, may not pose an obstacle for their potential use as QS inhibitors.

Another evaluated aspect on a selection of the plant extracts was the antibiofilm activity. Microbial biofilms are of great concern in management of bacterial and even fungal infections. Biofilm formation is characterized in part by the extensive production of exopolysaccharide (EPS) that results in the creation of a molecular network which confers facilitation of initial attachment of bacteria; enhanced resistance to antimicrobial agents and environmental stress; as well as formation of micro-colony structures [42]. It has also been reported that quorum sensing chemical signals (AIs) also regulate the production of EPS, which acts as a protective barrier for cells [33, 43] and enhances biofilm formation. Therefore, novel compounds that can inhibit quorum sensing, could also partly assist in management of biofilms and hence, in the control of bacterial infections. Even though these two extracts showed some antimicrobial effects, the methanol *Elaeodendron buchananii* extracts and 50% ethanol *Acacia gerrardii* used for this bio assay, did not show any antibiofilm formation effects at neither of the assayed doses of 1.0 and 0.5 mg/mL against *E*.*coli* Top 10 QS biosensor (*n* = 3). On the contrary, both seemed to promote biofilm formation (Fig 6). Seemingly the studied extracts could not be in possession of any lead compounds that could be targeting the inhibition of other sites of biofilm formation. One of such sites include the inhibition of diguanylate cyclases (DCGs) that is responsible for the production bis-(3’-5’)-cyclic dimeric guanosine monophosphate (c-di-GMP) in bacteria cell; a molecule that controls the biosynthesis of adhesins and exopolysaccharides associated with biofilm formation [44]. Some phytochemical compounds like N-[4-(phenylamino) phenyl]-benzamide have been reported to possess such activity [45]. Another possible target site for antibiofilm activity is dispersal of biofilms mainly by targeting the breakdown of the extracellular polysaccharide that makes up the extracellular matrix. Phytochemical compounds like norspermidine have been found to possess this property [46]. Therefore, at it could be concluded that the test extracts did not possess any of these activities and therefore could not be good candidates for management of biofilms related to this test isolate. However further work needs to be done other groups of bacteria to ascertain if indeed the extracts could be having any effects.

Both of the extracts of the selected plants seemed to exert cytotoxic effects on to MDCK-C7 cells, even at very lower doses (0.025 mg/mL). Only at the lowest assayed doses of 0.01 mg/mL the extracts resulted in cell viability greater than 50% (Fig 7). These extracts also seemed to have some interference on the MTT assay at higher doses (see S2 Fig). This is in keeping with previous results that have been reported medicinal plant extracts which tend to reduce the MTT reagent. For instance Peng *et al*., [47] did indicate that there was a significant reduction of MTT when it was incubated with luteolin and quercetin without any mammalian cells. These experimental results suggested that flavonoids reduced MTT in a concentration–time-dependent manner. Since such flavonoids may occur in the list of phytochemicals present in the assayed extracts, it is plausible to suspect that they may have led to the false apparent increase in cell viability as the result of the reduction of the MTT reagent especially at higher doses.

## Conclusion

In summary, the present work has led to gain knowledge to on the grounds of the use of traditional medicinal plants to manage bacterial infections in Africa. Our data clearly demonstrated that none of the addressed extracts possess exclusively potent antiquorum sensing properties mediated by AHL in Gram-negative bacteria. However, the majority of assayed plants exhibit antimicrobial properties. We have identified two plants for the first time to the best of our knowledge, namely, *Elaeodendron buchananii* and *Acacia gerrardii* that afforded ethanol and methanol extracts that seem to contain compounds with both antimicrobial and antiquorum sensing activities. However, future studies aimed to identify the individual pure chemical species present in these extracts will be necessary to validate our findings and shed further more light to the activity of the plant extracts that seemed to be promising.

## Supporting information

S1 Table The plant codes and extracted plant sample weight (grams) per medicinal plant with 50% ethanol as solvent and their plant codes. (DOCX)

S1 Fig Effect of methanol extracts of *Elaeodendron buchananii* Loes (P21) on the cell viabiliy of *E*. *coli* Top 10 QS biosensor strain agar-plate CFU determination. (DOCX)

S2 Fig MTT assay using high concentrations of *Elaeodendron buchananii* and *Acacia gerrardii* extracts. (TIF)
