# Supplementary figures and images for: Antiquorum sensing, antibiofilm formation and cytotoxicity activity of commonly used medicinal plants by inhabitants of Borabu sub-county, Nyamira County, Kenya
Source: PLoS One. 2017 Nov 1;12(11):e0185722. doi: 10.1371/journal.pone.0185722 (PMC5665492; doi:10.1371/journal.pone.0185722)

**S1 Fig.**


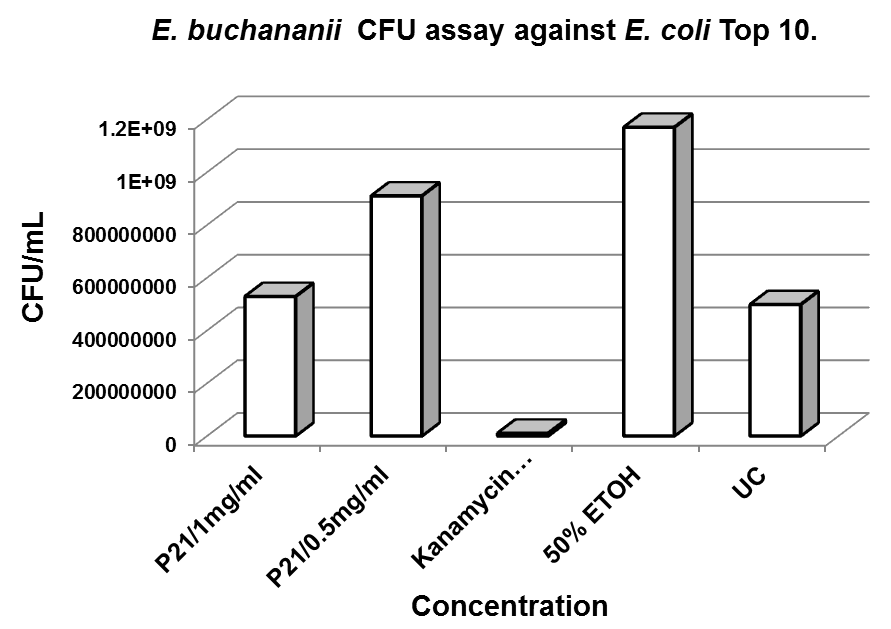

Supplement: S1 Fig — (DOCX) [file pone.0185722.s002.docx]

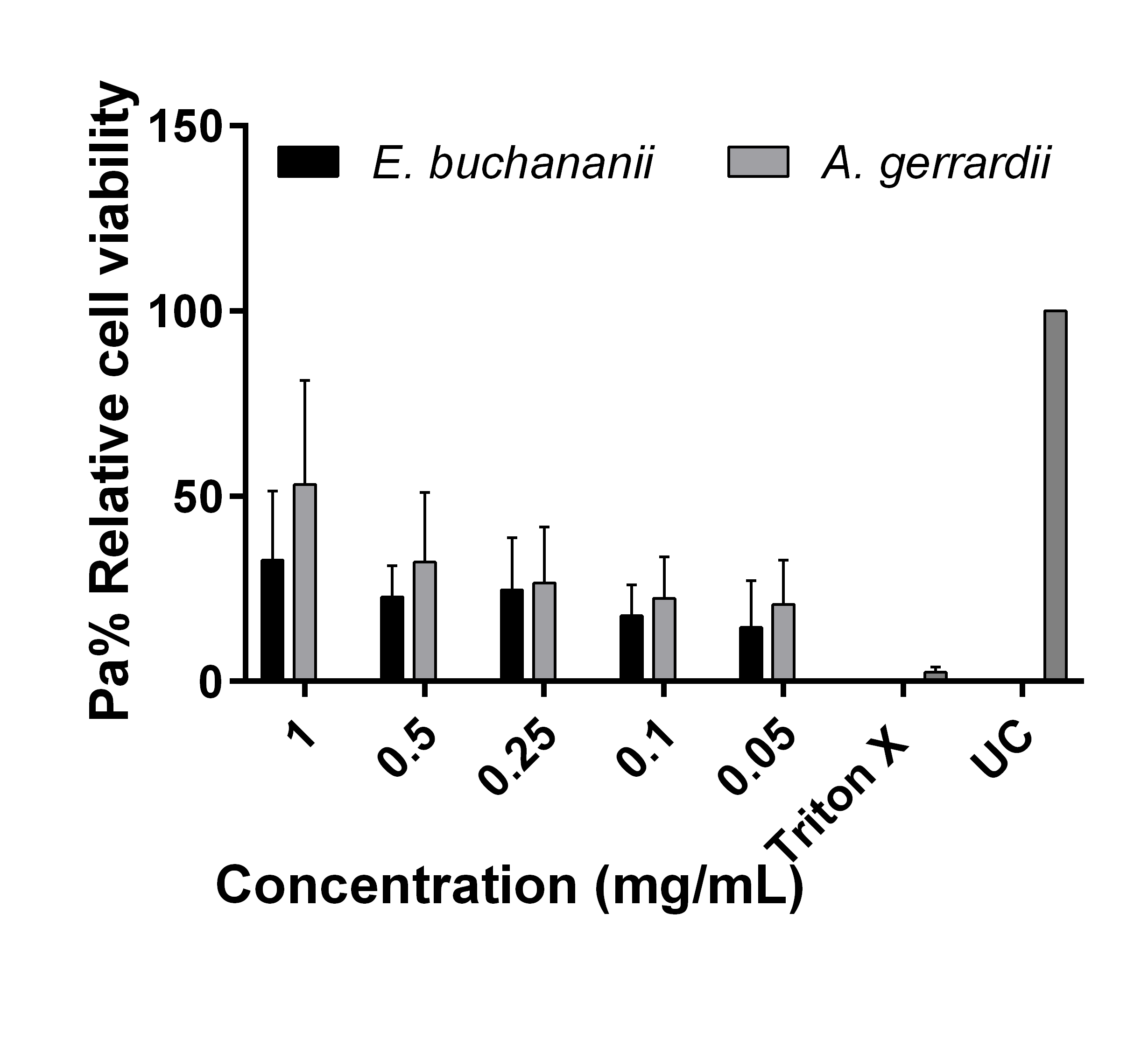

Supplement: S2 Fig — (TIF) [file pone.0185722.s003.tif]
